# Self-organizing spots get under your skin

**DOI:** 10.1371/journal.pbio.2004412

**Published:** 2017-12-20

**Authors:** Damian Dalle Nogare, Ajay B. Chitnis

**Affiliations:** Section on Neural Developmental Dynamics, Division of Developmental Biology, Eunice Kennedy Shriver National Institute of Child Health and Human Development, National Institutes of Health, Bethesda, Maryland, United States of America

## Abstract

Sixty-five years after Turing first revealed the potential of systems with local activation and long-range inhibition to generate pattern, we have only recently begun to identify the biological elements that operate at many scales to generate periodic patterns in nature. In this Primer, we first review the theoretical framework provided by Turing, Meinhardt, and others that suggests how periodic patterns could self-organize in developing animals. This Primer was developed to provide context for recent studies that reveal how diverse molecular, cellular, and physical mechanisms contribute to the establishment of the periodic pattern of hair or feather buds in the developing skin. From an initial emphasis on trying to disambiguate which specific mechanism plays a primary role in hair or feather bud development, we are beginning to discover that multiple mechanisms may, in at least some contexts, operate together. While the emergence of the diverse mechanisms underlying pattern formation in specific biological contexts probably reflects the contingencies of evolutionary history, an intriguing possibility is that these mechanisms interact and reinforce each other, producing emergent systems that are more robust.

Darwin concluded his book *On the Origin of Species* with the statement “from so simple a beginning endless forms most beautiful and most wonderful have been, and are being, evolved,” with which he invited us to reflect on how these elaborately constructed forms have all been produced by nature. At a time when little was known about genes, genetic regulatory networks, and their potential to regulate morphogenesis in the embryo, a central question was how pattern emerges from a situation for which no obvious prior pattern exists. Indeed, it seemed at least initially that such spontaneous generation of pattern somehow defied the known laws of physics and chemistry.

In 1952, just two years before tragically taking his own life, Alan Turing (1912–1954), the brilliant mathematician, computer scientist, and cryptanalyst, published his seminal paper “The Chemical Basis of Morphogenesis.” Just as Turing’s cryptographic insights provided new tools with which the Allied code-breakers at Bletchley Park could break the German enigma cypher, this paper provided a new mathematical framework to understand how chemical substances with near homogenous distributions could spontaneously form stable periodic patterns.

In what has become the paradigmatic case, Turing imagined a pair of freely diffusing substances, X and Y. In this system, X is able to catalyze both its own production as well as the production of Y, while Y is able to inhibit X. In addition, both are produced and degraded at some baseline rate. In these systems, X is typically referred to as the “activator” and Y as the “inhibitor.” Systems of this type, consisting of freely diffusing and reacting molecules, have come to be known as “reaction-diffusion” systems.

Turing showed that such systems, starting from near homogenous distributions, had the potential to form a variety of patterns—including oscillations and waves—through spontaneous interaction. However, it was the discovery of the formation of stable, periodic patterns in the concentration of these interacting substances (so-called “Turing patterns”) that remains the key breakthrough of this approach. Such patterns typically exploit differences in the diffusion range of the activator and the inhibitor as the basis for pattern generation. In fact, an important analytical insight to emerge from such reaction-diffusion systems was the discovery that if the concentration of the interacting agents is stable and homogeneous under conditions in which diffusion is not permitted but is expected to become unstable when diffusion is permitted, then the system of interacting agents would have the potential to spontaneously reorganize to form a wide range of possible stable patterns, including spots, stripes, and many complex patterns in between (Fig 1F–1H; see also Fig 4 in [1]). These types of symmetry-breaking instabilities that exploit diffusion to generate stable, nonhomogeneous steady states have come to be known as Turing instabilities.

**Fig 1:**
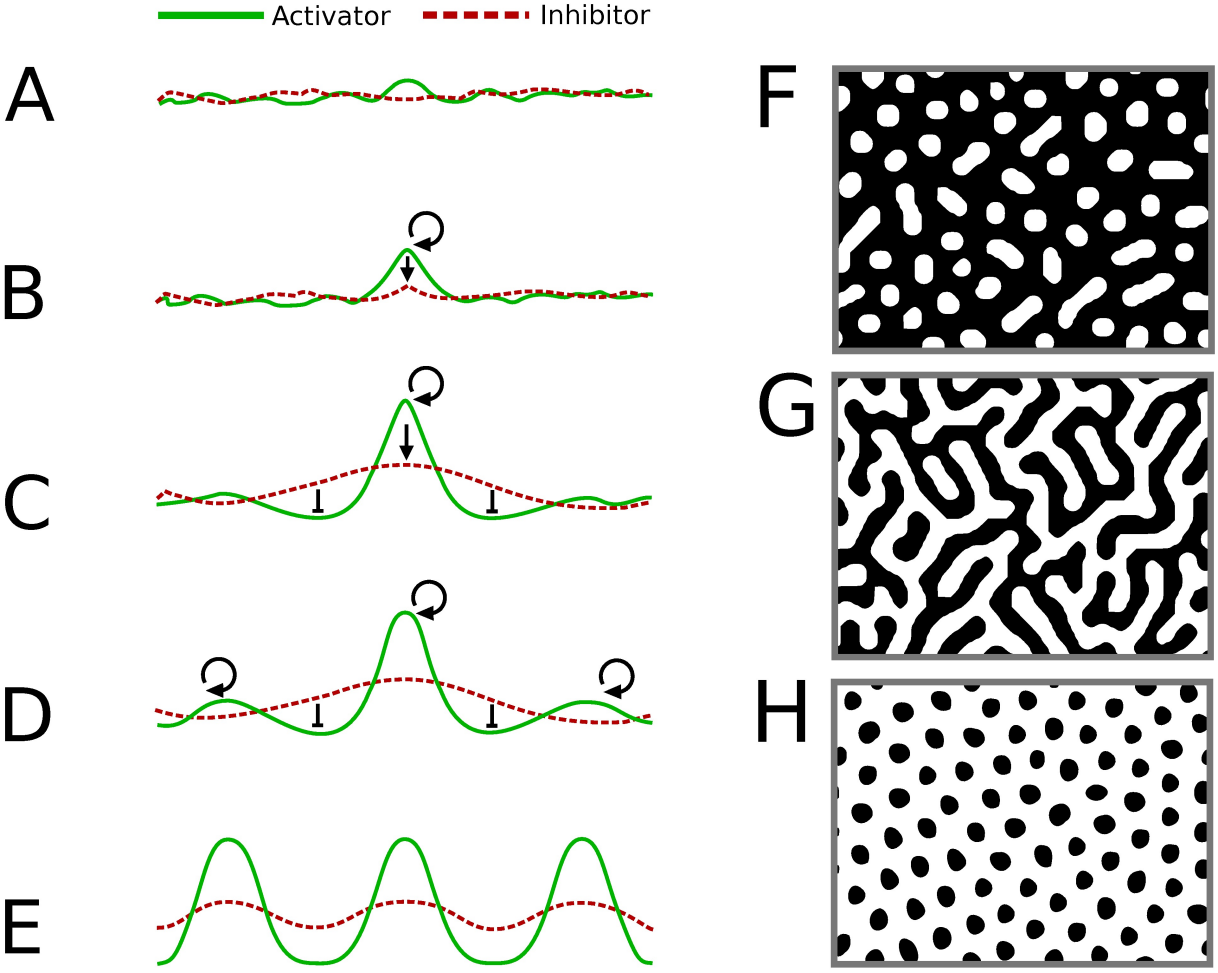
Spontaneous generation of pattern by short-range autocatalysis and long-range inhibition. The fundamental idea here can be intuitively understood by imagining a situation in which an inhibitor is capable of diffusing farther than an activator. In this situation, a small difference in the initial amounts of the activator and the inhibitor (A) can become amplified due to the self-catalyzing nature of the activator (B). This activity is relatively localized due to the short diffusion range of the activator. At the same time, production of the inhibitor will also increase at this location (C). As this inhibitor diffuses more rapidly to surrounding regions, it will have the effect of inhibiting the formation of more activator in more distant regions, while remaining insufficient to effectively overcome the autocatalytic production of the activator in the central region. In this manner, a single, stable peak can form (C). Further peaks can form at a distance, where the concentration of the inhibitor is sufficiently low (D, E). If the effective range of the rapidly diffusing inhibitor is comparable to the size of the field, then only one peak and a monotonic activator gradient will result. However, if the inhibitor’s effective range is small compared with the size of the field, then basal production of the activator would allow additional peaks of activator to build up outside the effective range of inhibitor. (F–H) Examples of patterns formed by a local activation–long range inhibition mechanism.

Turing’s idea that diffusion could make a stable chemical state unstable was innovative because diffusion is typically thought to have a homogenizing effect, as with, for example, the dispersion of an ink droplet in water. Turing demonstrated that the competition between activation by a slowly diffusing activator and inhibition by a more rapidly diffusing inhibitor would generate an instability that would result in the reorganization of the chemicals to form stable periodic patterns. He then suggested that the resulting heterogeneous distribution of such chemicals could serve as a prepattern for the subsequent differential morphogenesis or differentiation of cells that respond to such chemical “morphogens.” Nevertheless, in part because of the challenging nature of the mathematical analysis presented in his paper and the difficulty of mapping the abstraction of “reacting and diffusing chemicals” in the mathematical framework to the actual elements of complex biological systems, the implications and relevance of Turing’s paper was not broadly appreciated by developmental biologists at the time.

The biological relevance of “Turing patterns” eventually became more widely appreciated, at least in part, because of subsequent work by Hans Meinhardt and his mentor Alfred Gierer, who twenty years later published “A theory of biological pattern formation” [2]. This was followed by Meinhardt’s 1982 book *Models of Biological Pattern Formation* [3], in which a more accessible and intuitive explanation for such pattern-forming systems and their potential role in biology was provided. Though the equations described by Meinhardt are of the general reaction-diffusion type introduced by Turing, their theory had independent roots, and it emphasized how patterns emerge when local autocatalysis is coupled with long-range inhibition (see Fig 1A–1E).

Though patterning in a variety of biological contexts can be described in mathematical terms to be operating via Local Activation coupled with Long-range Inhibition (LALI), it has always been recognized that this abstraction can represent a wide range of regulatory relationships operating at many different scales and represent systems of interacting agents rather than, literally, a pair of interacting factors [3, 4]. Long-range inhibition could, for example, be achieved indirectly by the depletion of a rapidly diffusing substrate from neighboring domains as it is consumed by activator autocatalysis. Similarly, local activation could be achieved indirectly by mutually antagonistic systems, such that each system locally inhibits the function of its antagonistic system. Mathematical analysis and simulation of these different variations has helped reveal the full potential and define constraints within which different versions of such LALI patterning mechanisms could effectively operate in actual biological contexts [5].

From their initial historical role in providing a framework for understanding patterned growth and polarized regeneration in the fresh water polyp hydra [6], LALI reaction-diffusion mechanisms have been used over the years to provide a framework for understanding a wide range of biological pattern-forming events, including the generation of mammalian coat [7] and fish skin patterns [8], digit/nondigit patterning during chondrogenesis [9–11], feather bud and hair follicle patterns in the skin [1, 12], shell pigment patterns [13], and the pattern of rugae—or ridges—in the developing mammalian palate [14]. While the contribution of the LALI framework has now been accepted in understanding the emergence of pattern in many of the diverse biological contexts described above, its relevance remains a subject of active debate in some contexts, for example in the emergence of fish skin patterns [15–18]. Furthermore, it turned out to be misleading and inappropriate for explaining the emergence of early striped patterns in the *Drosophila*. Unfortunately, this historical failure contributed to a backlash and dismissal of the potential value of the LALI framework for years by many developmental biologists. These problems notwithstanding, our understanding of the mechanisms by which cells interact in diverse biological contexts now illustrates how “local activation and long-range inhibition” can be achieved by a variety of mechanisms that do not always involve the actual diffusion of “activator” and “inhibitor” molecules; instead, cells can influence the fate and behavior of their neighbors by a variety of alternate molecular, cellular, or physical mechanisms [12]. As individual abstract mathematical models can account for pattern generated by diverse underlying physical mechanisms, it has become important to develop mathematical and experimental approaches to distinguish between these alternate mechanisms [5] and to understand how they might work together to determine a robust patterning system. The next few sections discuss recent studies that illustrate how diverse molecular, cellular, and physical mechanisms operate at different scales to generate periodic patterns during animal development and how the interaction between these mechanisms might contribute to robust pattern formation.

The distinctive stripes of the zebrafish, a popular model organism, arise in part because of interactions between xanthophores (yellow pigment cells) and surrounding melanophores (black pigment cells) [8]. Each of these types of pigment cells mutually inhibits the local accumulation of the other cell type. At the same time, long-range interactions allow xanothophores to promote the survival of melanophores. The potential of such interactions to determine self-organization of spots or stripes of black and yellow cells has been effectively demonstrated with simulations that represent these interaction as a reaction-diffusion system [8]. In these simulations, xanthophores locally promote their own accumulation by inhibiting the accumulation of melanophores, while at a distance they inhibit their own accumulation by promoting survival of melanophores. In vivo, however, critical interactions between the cells that influence their eventual distribution are not necessarily determined by diffusible factors. Instead, they are determined, at least in part, by cytoplasmic processes extended by pigment cells and their precursors to influence the movement and/or survival of neighbors [19, 20]. While diffusion-based models effectively predict emergent patterns in this context, they do not accurately represent the influence of pigment cells or their precursors on neighboring cells as a function of distance. Diffusion results in a graded influence, which is strongest at its source and rapidly decreases as a function of distance. Therefore, it is difficult to use diffusion to model the influence of filopodia, whose influence on neighboring cells via direct contact is maximal at the tip of these protrusions. To model systems that include such nonlocal interactions, Murray introduced the use of a kernel function, which quantifies both activating and inhibitory effects of a neighboring cell as a function of its distance [21]. This approach, extended recently by Shigeru Kondo, has been used to develop a kernel-based Turing (KT) model for studying mechanisms of biological pattern formation [22]. This extended framework allows the modeling of interactions in a manner that is agnostic about the specific physical mechanisms that determine interactions between close and more distant neighbors. The KT framework not only recapitulates patterns predicted by a diffusion-based LALI framework but is far more flexible, and many constraints originally thought to be essential for pattern formation in diffusion-based systems no longer apply. For example, Kondo shows how an inverse LALI system with a shorter inhibitor and longer activator range also has patterning potential when specific conditions are met.

While the discovery of cell–cell interactions via cytoplasmic processes has led to an extension of Turing’s theoretical framework as discussed above, precisely how interactions between pigment cells and their neighbors contribute to emergent striped patterns remains a subject of active investigation [8, 19, 23–25]. The analysis of zebrafish mutants has revealed the potential role of water channels, gap junction proteins, solute carriers, transporters, immunoglobulin superfamily members, tight junctional proteins, and several ligands, receptors, and downstream transcription factors in the self-organization of stripe patterns in zebrafish [24]. These discoveries—along with a detailed analysis of pigment cell movement, morphology, and dynamic interactions with time-lapse imaging [8, 25–29]—pose fresh challenges to those hoping to understand pattern formation in the context of Turing patterning mechanisms [17].

While the Turing framework has been successfully modified to account for pattern formation by a variety of cell–cell signaling mechanisms, the physical movement of cells can play an equally important role in many contexts. In this context, response to a chemoattractant can promote formation of cell aggregates, while the accompanying depletion of cells in the surrounding area makes it less likely that new aggregates will form close by (Fig 2A). Such a patterning system could be described as one with local accumulation accompanied by long-range inhibition by depletion of a substrate (in this case, cells). The question of whether a periodic pattern of cell aggregates emerges in response to a prepattern of morphogens established by a reaction-diffusion mechanism or directly by movement of cells has been a central puzzle in determining how the periodic pattern of hair follicles emerges in the mammalian skin, as discussed in the next section.

**Fig 2:**
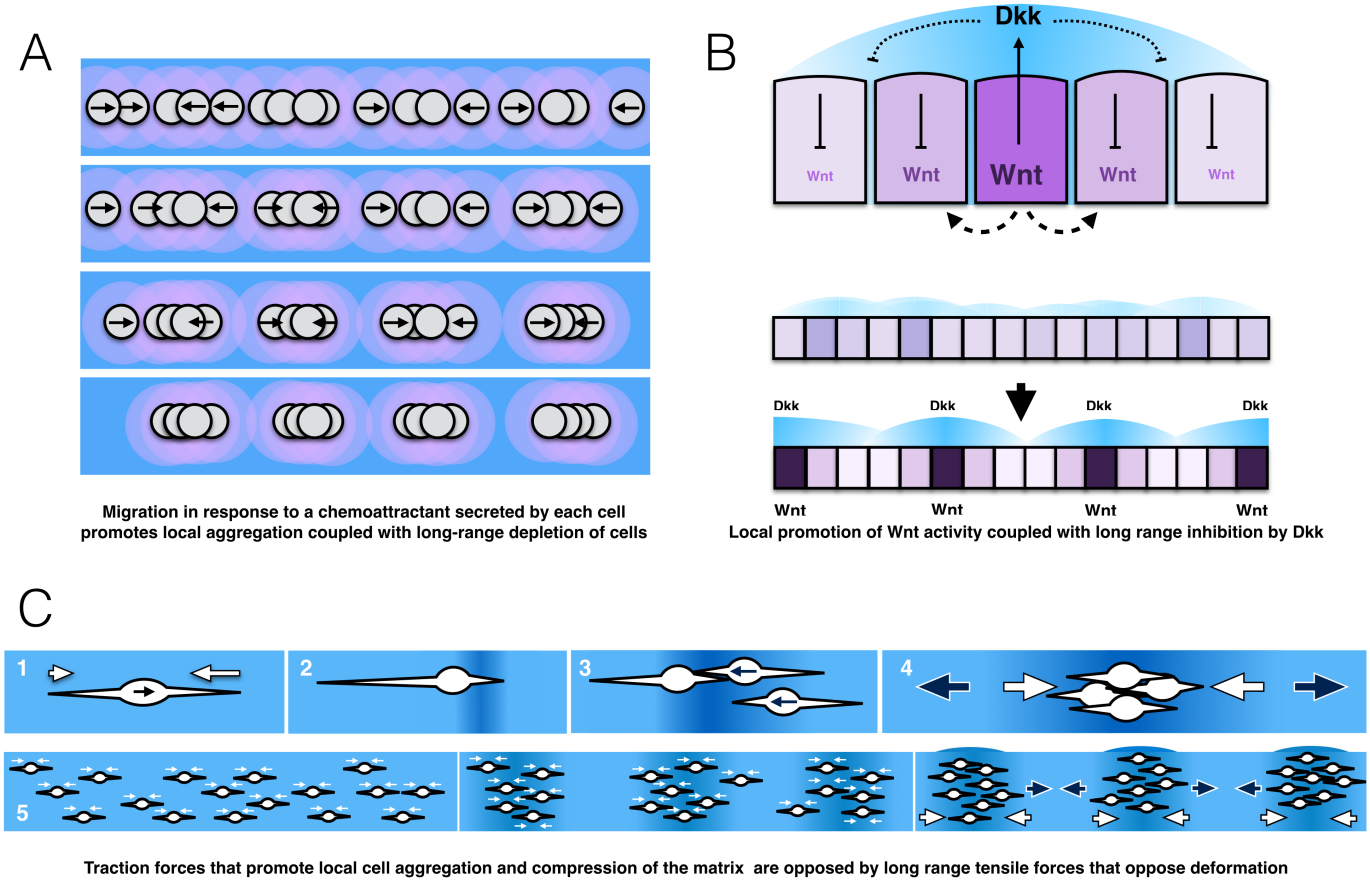
Cellular, molecular, and physical models of pattern formation via local activation and long-range inhibition. (A) Periodic patterning of cell clusters determined by local aggregation in response to a chemotactic factor. Cells move toward each other as they secrete a chemotactic factor (pink). Small clusters become stronger sources, locally promoting formation of larger aggregates. However, as cells move to become part of larger aggregates, they are depleted in surrounding regions, inhibiting the formation of adjacent clusters. (B) Periodic formation of Wnt signaling centers by local activation and long-range inhibition of Wnt signaling. Top panel: Wnt diffuses a short distance to activate Wnt signaling and locally drive its own expression. Wnt signaling also drives expression of the Wnt inhibitor Dkk, which is assumed here to diffuse more rapidly to surrounding cells, where it inhibits Wnt signaling. Bottom panel: An initially unpatterned row of epidermal cells with varying levels of Wnt activity becomes patterned into periodic peaks of Wnt activity by the action of the LALI mechanism. Blue clouds represent fields of diffusing Dkk protein. The darker shades of purple represent cells with higher Wnt activity. (C) Periodic clustering of cells by the balance of traction forces that locally promote cell aggregation and long-range forces that resist deformation of the matrix. (1) As a cell adheres and pulls on the matrix (small white arrows), a tug-of-war of traction forces determines the direction in which the cell moves (black arrow in cell). (2) The traction forces compress the surrounding matrix (darker blue matrix). (3) Compression of the matrix increases the density of adhesive sites, in addition direct cell contact promotes local cell movement toward this site (black arrows in cells). (4) Local traction forces that promote compression and cell aggregation (white arrows) are balanced by long-range elastic forces (black arrows) in the matrix that oppose matrix deformation. (5) These physical mechanisms operating in an initially unpatterned field of cells can lead to the spontaneous formation of periodic cell clusters. Dkk, Dickkopf; LALI, local activation coupled with long-range inhibition.

The development of the periodic pattern of hair follicles in the skin of a mouse embryo is marked by two correlated patterning events, one in the overlying epidermis and another in the underlying dermis, which contains mesenchymal cells [30]. The first indication of pattern in the epidermis is the appearance of the periodically distributed expression of *dkk4* encoding a secreted Wnt antagonist. This is followed by closer packing of cells and the thickening of the epidermis in the spots with *dkk4* expression to form placodes. Epidermal placode formation is accompanied by the expression of *dkk1* in adjacent underlying mesenchymal cells, which aggregate to form dermal condensates. Expression of the Wnt inhibitor Dickkopf (Dkk) in both the epidermis and the underlying dermis is dependent on Wnt signaling, which suggested that the pattern of Wnt-dependent Dkk expression is determined, at least in part, by a reaction-diffusion system. In such a system, Wnt activity would locally promote the formation of placodes or mesenchymal condensations, while Dkk secreted in response to local Wnt activity would determine long-range inhibition of this process (Fig 2B). Simulations based on such an assumption show how a reaction-diffusion system could determine the distribution of hair follicles and, furthermore, how their distribution would become sparser as Dkk was increased [30]. Though these predictions were borne out by manipulations in which Dkk2 expression was artificially induced, an examination of all the elements involved in this patterning system revealed that the actual system is more complicated. First, there was no evidence that Wnt was locally promoting its own expression. Second, there was evidence that cells were interacting via several additional signaling pathways, including Fibroblast Growth Factor (FGF), Bone Morphogenetic Protein (BMP), Transforming Growth Factor Beta (TGFβ), and Ectodysplasin A receptor (EDAR), that mediate interactions between cells. This made it difficult to determine which of these various signaling systems contribute to establishment of the prepattern. Furthermore, it remained possible that an independent self-organizing process resulting from cell movement and aggregation was determining patterning of underlying dermal condensates.

A paper by Glover et al. [31] now helps define the signaling factors that interact to establish a prepattern of Wnt signaling in the epidermis. Mathematical analysis of Turing reaction-diffusion systems had shown that for stable patterns to emerge, the reacting substances must have a relatively short half-life. Based on this constraint, the authors did a transcriptome-wide screen to determine which transcripts have a short enough lifetime to qualify as potential components of an effective reaction-diffusion patterning system. This analysis confirmed that components of the Wnt, FGF, and BMP signaling pathways could contribute to establishing a prepattern, while components of the EDAR and TGFβ pathway had longer lifetimes and were likely to help maintain prepattern established in an earlier step.

Then by either activating or repressing each of these signaling systems, the authors determined the regulatory relationships between the signaling pathways. While this analysis confirmed that activation of some pathways stimulated expression of their own inhibitors, there was no evidence for direct positive feedback. Instead, each pathway displayed prominent self-inhibition rather than self-activation. Because the regulatory relationships did not correspond in a simple way to the topology of a classic Turing mechanism, the authors took a mathematical approach to determine whether the interactions had the potential to produce a periodic pattern. In this approach, candidate genes were put into groups based on whether they promote either Wnt, FGF, or BMP signaling or inhibit BMP or Wnt signaling. Then, a matrix representing whether genes in one group activate or inhibit genes in another group was developed. Instability analysis using this matrix showed that a small perturbation in the system would be expected to grow if each group included at least one member that was diffusible. Because each group did in fact include a diffusible factor, conditions for “Turing instability” were met, and the system was predicted to have the potential to form stable periodic patterns.

Having established that interactions mediated by Wnt, FGF, and BMP signal have the potential to establish a periodic pattern of Wnt signaling by a reaction-diffusion mechanism, the authors go on to show that the resulting prepattern of Wnt activity in epidermal cells determines local aggregation of underlying mesenchymal cells. The periodic pattern of Wnt activity in the epidermal cells is associated with local epidermal thickening and the formation of epidermal hair placodes. These epidermal cells also become a local source of FGF20, which determines directed migration of underlying mesenchymal cells to form condensates at the source of FGF20. As mesenchymal cells form condensates, they also become a source of BMP4. However, BMP signaling is kept in check by BMP antagonists expressed by the mesenchymal condensate and the overlying epidermal cells. Investigation of the role of BMP signaling in this context showed that exposure to BMPs destabilizes dermal condensates. Together their observations suggest that while FGF signals promote local aggregation, BMP signals limit this process and that strong FGF signaling coupled with attenuated BMP signaling coordinates formation of the underlying periodic pattern of mesenchymal condensates.

While FGFs, BMPs, and BMP antagonists, expressed in response to the establishment of a prepattern of Wnt activity in epidermis, normally coordinate aggregation of underlying mesenchymal cells, the question remained whether patterned aggregation of mesenchymal cells can emerge independent of prepatterning signals from the epidermis. When mesenchymal cells were exposed to a combination of FGF ligand and BMP inhibitor in the absence of the epidermal prepattern, they autonomously reorganized to form a periodic pattern of condensates. However, there were several differences in the behavior of mesenchymal cells and the way the pattern emerged when the mesenchymal cell behavior was determined autonomously. Normally, directed migration is only observed in mesenchymal cells that are relatively close to epidermal cells that are a focal source of FGF signals. In contrast, when all the mesenchymal cells were artificially exposed to relatively high FGF coupled with low BMP signaling, all the mesenchymal cells participated in directed migration. In addition, there was a key difference in where mesenchymal condensates first formed with respect to the tissue boundary. A reaction-diffusion patterning system based on diffusible signals favors self-organization of active zones adjacent to tissue boundaries. This is because as cells compete to form stable active signaling centers in the context of local activation and long-range inhibition, cells at the tissue edge are at an advantage because they receive no inhibitory signals from cells outside the tissue boundary. Consistent with this expectation, the authors show that mesenchymal condensates form adjacent to tissue boundaries when their organization is determined by the overlying epidermal prepattern. On the other hand, when cell aggregation autonomously determines self-organization of a periodic pattern, locations at a tissue boundary are at a disadvantage because they have fewer cells in the surrounding area to draw on to form the initial aggregates. Consistent with this expectation, when mesenchymal condensates formed autonomously in the absence of epidermal prepattern, the authors showed that condensates now formed at some distance from the tissue edge rather than immediately adjacent to it.

In the previous section, cell movement associated with cell aggregation was described as an abstract process that has the potential to determine a periodic pattern of cell distribution as local aggregation is accompanied by depletion of cells from surrounding areas. However, cells generate substantial traction forces as they migrate. In 1983, Oster, Murray, and Harris [32] described how the traction forces associated with cell migration may themselves have the potential to generate periodic patterns of cell aggregation (Fig 2C). As cells extend processes that adhere and pull in different directions, a tug-of-war ensues, with cells eventually moving in the direction with the strongest pull and most effective adhesions. However, the pull of a migrating cell also draws the surrounding matrix closer, changing the adhesive properties of the matrix and the migratory behavior of surrounding cells. As a cell becomes the center of a local contractile force, compression of the surrounding matrix increases the local density and alignment of adhesive sites, which can bias migration of surrounding cells toward the center of the contractile force. In addition, the local traction forces can themselves passively pull surrounding cells closer. As the density of cells drawn together increases, so does the local contractile force, promoting more aggregation. However, elastic forces that resist distortion of the matrix material oppose the traction forces that promote the local compression of the matrix and aggregation of cells. Ultimately, at some distance from the growing cell condensate, stiffness of the matrix equilibrates with the contractile force, limiting the range of influence of the cell condensate. In this manner, as in the self-organizing LALI systems described above, contractile forces that promote cell aggregation at a short range are opposed by stiffness of the matrix that serves as a long-range inhibitor of cell aggregation. Oster et al. developed mathematical models based on the considerations described above to illustrate how the balance forces determined by cell migration and/or the traction forces exerted by cells on the surrounding matrix have the potential to generate the periodic pattern of cell condensates observed in the skin during the formation of mammalian hair follicles and chick feather buds [32].

Now a paper published by Shyer et al. [12] shows how mechanical forces within the dermis may indeed be responsible for establishing the initial pattern of dermal cell condensates in the avian skin during the formation of feather buds by a mechanism like the one described above. The avian embryonic skin also consists of a superficial sheet of epithelial cells attached by a basement membrane to a deeper layer of mesenchymal cells. Over a period of approximately 48 hours, the relatively uniform tissue bilayer transforms into one characterized by regularly spaced multicellular aggregates. Each cell aggregate is associated with characteristic foci of Wnt activity in the overlying epidermis and *bmp2* expression in the underlying dermis. Though the initial morphogenetic and cell-signaling events associated with feather bud and hair follicle formation in the avian and mammalian skin appear to be remarkably similar, the Shyer et al. [12] and Glover et al. [31] studies come to very different conclusions about how pattern is initiated in these two developmental contexts. As described above, Glover et al. conclude that a periodic pattern of Wnt signaling first established by a molecular reaction-diffusion mechanism in the overlying epidermis helps establish foci of FGF signaling that subsequently direct migratory behavior of mesenchymal cells in the underlying dermis. Shyer et al., on the other hand, found that Wnt activity, which also initiates expression of genes that determine follicle fate in the avian skin, accompanies rather than precedes the earliest morphological changes associated with follicle formation. They also observe that there is inherent tension present in the embryonic skin; when a piece of it is excised, it spontaneously shrinks and adopts a tissue architecture like that of feather bud primordia with bunched epithelial cells, aggregated mesenchyme, and a buckled basement membrane. As in the mammalian skin, they also demonstrate that formation of cellular aggregates is not necessarily dependent on Wnt signaling in the epidermis. Importantly, however, in ex vivo culture experiments in which they systematically alter either the contractility or stiffness of the tissue, they show how the balance of contractile forces in the dermis opposed by stiffness of the tissue determines the self-organization of multicellular aggregates. Their observations suggest that mesenchymal cells in the dermis have a propensity to aggregate, and as they do, they draw in more cells. However, this potential to aggregate is opposed by the stiffness of the tissue. Under conditions in which the substrate is either too stiff or too soft, no pattern emerges. However, at intermediate stiffness, regularly spaced aggregates form with spacing increasing as a function of stiffness. Similarly, dramatically reducing or exaggerating contractility prevents self-organization of cellular condensates, which only form with intermediate levels of contractility.

What then is the role of the foci of Wnt activity in the embryonic avian epidermis, and how do these foci emerge? As multicellular condensates form in the dermis, they compress overlying epidermal cells to which they are mechanically coupled via the basement membrane. Remarkably, this compression releases β-catenin, which translocates to the nucleus to promote Wnt activity in the epidermal cells. This, in turn, initiates the follicle primordium gene expression program, including, eventually, the expression of *bmp2* in the underlying dermal cells. In this manner, while mechanical forces appear to initiate a pattern of dermal condensates in the dermis, they also initiate Wnt signaling in the overlying epidermis, which reinforces and/or refines the periodic pattern of developing feather bud follicles.

We have come a long way since Turing first described a mathematical framework for understanding how periodic patterns can emerge spontaneously in nature. From a time when the relevance of this theoretical framework was unclear, we are now beginning to recognize self-organizing systems operating at many scales via diverse molecular, cellular, and physical mechanisms. From an emphasis on trying to disambiguate which of these diverse mechanisms plays a primary role in a specific developmental context, we are beginning to discover that these patterning mechanisms may, in at least some contexts, operate together. The broader lesson from recent studies of hair follicle and feather bud patterning is that information from these diverse patterning systems moves in both directions to interact and reinforce each other. While the existence of multiple interacting mechanisms underlying pattern formation probably reflects the contingencies of evolutionary history, it is possible that they work together to produce emergent systems that are more robust. The integration of this diversity and complexity into the theoretical framework has revealed how many constraints thought to be important in a diffusion-based mechanism need not apply in natural systems operating via alternate mechanisms. Together, recent studies reveal how interactions between theoretical and experimental biologists and the growing recognition of the relevance of theoretical patterning mechanisms, whose foundations were laid more than half a century ago, promise a rich future and deeper understanding of pattern formation in animal development.

## Abbreviations

BMP: Bone Morphogenetic Protein
DKK: Dickkopf
EDAR: Ectodysplasin A receptor
FGF: Fibroblast Growth Factor
KT: Kernel-based Turing
LALI: Local Activation coupled with Long-range Inhibition
TGFβ: Transforming Growth Factor Beta
